# Matched and Measured: An In-depth Look at the Interventional Cardiology Fellowship Match—Fellows’ Perspective

**DOI:** 10.1016/j.jscai.2026.105454

**Published:** 2026-06-11

**Authors:** Naseer A. Khan, Najam U. Saqib, Eva Meunier, Dean J. Kereiakes, Luay Alalawi, Said Hajouli, Muhammad Saleem, Abubakar Nazir

**Affiliations:** a Department of Cardiovascular Medicine, University of Cincinnati, Cincinnati, Ohio; b Department of Cardiovascular Medicine, University of Chicago-Endeavor Health, Chicago, Illinois; c Department of Cardiovascular MedicineThe Christ Hospital, Cincinnati, Ohio; d Department of Cardiovascular Medicine, The Wright Center for Graduate Medical Education, Scranton, Pennsylvania; e Department of Medicine, The Jewish Hospital-Mercy Health, Cincinnati, Ohio

**Keywords:** Fellowship Administrators in Cardiovascular Education and Training, interventional cardiology fellowships, National Resident Matching Program match

## Abstract

**Background:**

In July 2024, the interventional cardiology (IC) fellowship match transitioned to the National Resident Matching Program (NRMP) to streamline recruitment and ensure transparency. Our prior analysis explored the program directors’ (PDs) experiences, revealing positive perceptions despite initial concerns. This current study addresses a critical limitation of the previous work by incorporating the applicants’ perspective. The aim of this study is to investigate perceptions of fellows who participated in the IC fellowship for the NRMP match cycles.

**Methods:**

This descriptive study utilized data from publicly available NRMP and Electronic Residency Application Services along with our survey to evaluate the IC fellowship match from the fellows’ perspective. An anonymous online survey was distributed to fellows to gather quantitative and qualitative data. Survey topics included questions about interviews, ranking, and factors influencing rank lists, and satisfaction with the new match system.

**Results:**

An anonymous national fellow survey was completed by 38% (N = 96) of IC fellows, most of whom were applicants to the 2026-2027 IC fellowship match. Seventy percent applied to >30 programs, with the majority receiving >15 invitations. Rank list depth varied, although most applicants ranked multiple programs, most commonly 6 to 10 (30%) or >15 (28%). Prematch offers were rare (13%), and 78% of applicants preferred training at external institutions, whereas the majority preferred virtual interviews. Qualitative responses emphasized procedural volume, program reputation, mentorship, and culture as key drivers of ranking decisions, whereas challenges included limited program information, inconsistent communication, uncertainty around prematch activity, and stress.

**Conclusions:**

The transition to the NRMP match for IC fellowships presented both successes and challenges from the fellows’ viewpoint, complementing the PDs’ perspectives documented previously. Our study helps highlight challenges in future IC match.

## Introduction

In July 2024, the interventional cardiology (IC) fellowship recruitment process was formalized by transitioning to the NRMP, a move advocated by the American College of Cardiology and the Society for Cardiovascular Angiography & Intervention (SCAI). The intent was to ensure a transparent, standardized, and equitable recruitment process for all stakeholders. Although a preliminary analysis offered insights from the perspective of PDs, this paper aims to characterize IC fellows’ experiences and perceptions of the NRMP match, identifying key challenges, opportunities, and interventions.

## Methods

NRMP match outcomes, Electronic Residency Application Services recruitment metrics, and data from an anonymous online survey of fellows who participated in the interventional cardiology (IC) match were utilized for this study. The survey was conducted between November 2025 and January 2026, following completion of the 2025-2026 match cycle, to capture postmatch applicant perspectives.

A structured, anonymous online survey consisting of 13 questions was developed to assess applicant experiences across multiple domains, including applicant status, application behavior, interview experience, rank list characteristics, prematch participation, training location preference, decision-making factors, perceived challenges, interview format preference, satisfaction with match transparency, and suggested improvements. The survey included both closed-ended (multiple choice and categorical) and open-ended questions to capture quantitative and qualitative data.

The survey was distributed via multiple channels, including social media platforms, direct outreach through PDs and program coordinators, and through the American College of Cardiology Fellowship Administrators in Cardiovascular Education and Training network, to maximize reach among applicants and fellows. Participation was voluntary, and responses were collected anonymously.

Most respondents were applicants to the 2026-2027 match cycle, with a smaller proportion comprising current fellows who had participated in prior cycles; however, a cycle-specific breakdown was not collected. Survey questions were reviewed by the study team to ensure clarity and content validity. Quantitative data were analyzed using descriptive statistics, whereas qualitative responses underwent thematic analysis. NRMP and Electronic Residency Application Services data were used in aggregate form, consistent with confidentiality policies. Survey questions are provided in the Appendix.

## Results

A total of 96 individuals (about 38% of the total applicants) completed the national survey, most of whom were applicants to the 2026-2027 IC fellowship match. In the last 2 NRMP match cycles for IC fellowships, participation remained relatively stable. In 2024, 152 fellowship programs joined the match, collectively offering 322 positions to 246 applicants, of whom 239 successfully matched. In 2025, the number of participating programs increased slightly to 153, although the total positions offered decreased to 307 and 244 applicants entered the match, with 236 securing positions. Among the applicants who applied for the 2026 match, 236 (97%) secured a position. There were 8 applicants who did not match, and 71 positions remained unfilled across all the IC fellowship programs.^1^

Survey responses from the NRMP match cycle reflected a broad and competitive application landscape. Most applicants applied extensively, with interview activity also demonstrating a similarly high volume (Table 1).

**Table 1:** Application and interview activity among IC fellowship applicants.

Category	N = 96
Applications submitted	
>30 programs	70%
20-30 programs	14%
10-20 programs	8%
<10 programs	8%
Interview invitations received	
>15 interviews	40%
11-15 interviews	23%
6-10 interviews	18%
1-5 interviews	19%
Interviews attended	
>15 interviews	36%
11-15 interviews	20%
6-10 interviews	23%
1-5 interviews	21%

Rank list behavior varied, although most applicants ranked multiple programs. Prematch was infrequent although this might not be a true reflection of reality as many candidates who accepted prematch might not have responded. Most applicants preferred training at an external institution rather than remaining internal at their general cardiology fellowship program (Table 2).

**Table 2:** Ranking behavior, match dynamics, and training preferences.

Category	N = 96
Rank list length	
1-5 programs	23%
6-10 programs	30%
11-15 programs	19%
>15 programs	28%
Prematch activity	
Offered/accepted	13%
Not offered/not accepted	87%
Training location preference	
Prefer external institution	78%
Prefer to stay internal	22%

Preferences regarding interview format showed a clear distribution with most preferring virtual interviews and the overall satisfaction with the transparency in the application process was mostly positive. Qualitative responses revealed consistent themes influencing applicants’ ranking decisions (Table 3).

**Table 3:** Interview format preferences and satisfaction with transparency.

Category	Percentage/details
Interview format preference	
Virtual	48%
Hybrid	30%
In-person	28%
Process transparency satisfaction	
Satisfied	52%
Dissatisfied	40%
Prefer not to answer	8%
Key qualitative themes	
Driving factors	Procedural volume and complexity; program reputation; mentorship; geographic factors; coronary/peripheral/structural exposure; program culture
Challenges	Limited program information; inconsistent communication; prematch uncertainty; overall Match stress
Suggested improvements	Eliminate prematch; clearer case volume disclosure; early Match participation confirmation; visa transparency; signaling system; expanded virtual interviews

## Discussion

This evaluation highlights the experiences of IC fellows during the NRMP match, providing a necessary counterpoint to the PD-focused analysis previously conducted. This recent trend regarding unfilled fellowship positions highlights a critical inflection point for the IC workforce and reveals 2 consistent trends: nearly all applicants are successfully placed, reflecting the reliability of the match process, and there continues to be a surplus of fellowship positions relative to the number of candidates. Although the number of applicants remained relatively stable across recent cycles (246 applicants for 322 positions in 2025-2026; 244 applicants for 309 positions in 2026-2027), the imbalance between available fellowship positions and applicants highlights ongoing challenges in workforce planning for IC. Rather than reflecting a decline in trainee interest, this mismatch underscores the need to align fellowship capacity with the pool of qualified applicants.

The results of our national fellows’ survey underscore the increasingly competitive dynamics of the IC fellowship match. Most applicants pursued an extensive and broad application strategy, and this approach was also paralleled by interview activity. Many applicants expressed a preference for training at external institutions, underscoring the value placed on diverse educational environments. Perceptions of transparency in the application process were mixed, with just over half reporting satisfaction. Qualitative responses provide important context to applicant decision-making, revealing that procedural volume and complexity, together with program reputation, were the most influential factors guiding rank list behavior. Some suggestions from the fellows to improve the match process included eliminating prematch pathways, enhancing transparency in case volumes and training structures, clarifying visa sponsorship policies, and adopting signaling systems. Although virtual interviews offer greater scheduling flexibility and reduce the travel and financial burden for applicants, our previous program director survey data suggested that some programs perceived candidates to be less engaged in the virtual format, raising concerns about the potential inefficiency and resource expenditure from the program’s perspective. The availability of more positions than candidates could be seen as an opportunity to expand access and diversify the workforce. Although having more fellowship positions than applicants could allow for expansion and diversification of the workforce, persistent systemic challenges such as application burden, limited transparency, and stress associated with the match may continue to discourage some trainees from pursuing IC. Lack of consistency with some programs either not participating in the match or offering out-of-match positions to the applicants was also a big concern for the applicants. Applicants also highlighted concerns about the timeline, describing the process as too drawn out and suggesting either more time to prepare applications or a separate subspecialty timeline distinct from the general fellowship match.

To place our findings in a broader context, prior studies evaluating applicant perspectives across residency and fellowship match processes, particularly in procedural and surgical specialties, have identified several consistent themes.^2^ Applicants frequently prioritize procedural volume, program reputation, mentorship, and institutional culture when formulating rank lists. Similar to our findings, these studies also report challenges related to limited program transparency, high application and interview burden, inconsistent communication, and significant stress associated with the match process. Our results are consistent with these observations and further extend the literature by focusing specifically on the IC match following its transition to the NRMP.^2^ The strong emphasis on procedural complexity and training exposure in our cohort reflects patterns seen in other procedure-intensive specialties, whereas concerns regarding prematch activity, unclear program participation, and variability in communication highlight unique issues associated with this evolving match structure.^2^

The SCAI Match Task Force identified several key factors that might dissuade trainees from pursuing this specialty. Some occupational and lifestyle factors also contributed to this trend.^3^ The use of ionizing radiation in the cardiac catheterization laboratory for coronary angiograms and intervention adversely impacts both the patients and medical staff and can lead to long-term health effects including certain forms of cancer by interacting with and altering cellular DNA.^4^ In addition, the demanding nature of call schedules coupled with high rates of burnout and concerns about long-term career viability, make the specialty less attractive to new physicians. Some barriers to physicians entering cardiology and specifically into IC may include the years of training required, work/life imbalances, and increasing administrative burdens.^5^ These findings highlight that workforce sustainability requires more structural changes to improve safety, wellness, and career longevity. In addition, lower Medicaid payments also impact hospitals.^6^ This negatively impacts program development and reimbursement for employed physicians (Central Illustration).

**Central Illustration:**
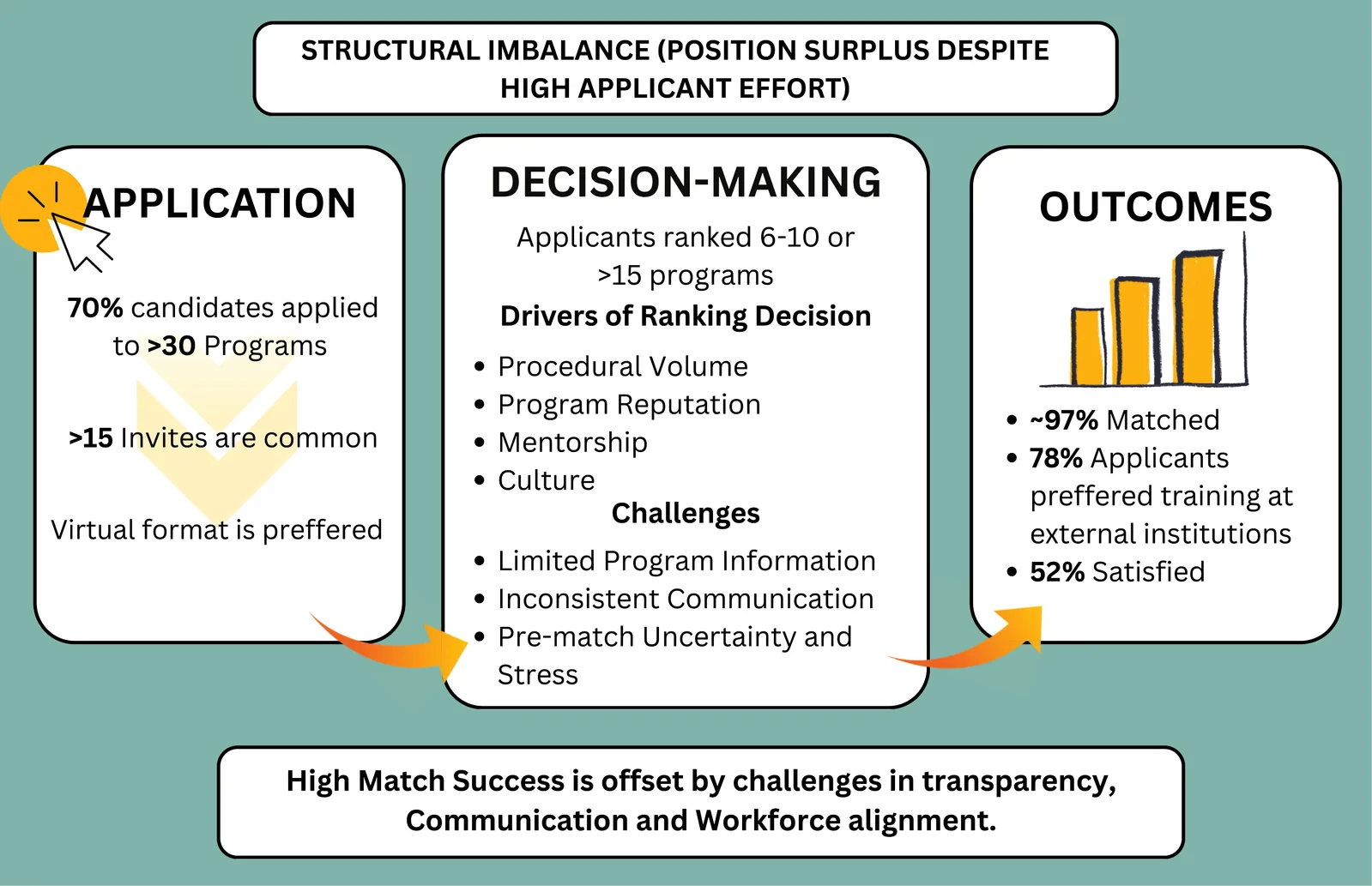
**Interventional cardiology fellowshipmatch.**

### Current challenges and future recommendations

The current mismatch between the number of IC fellowship positions and applicants reflects a broader set of structural challenges that extend beyond the match itself. Moving forward, consistent match participation, enhanced transparency, and elimination of prematch pathways are essential to restoring fairness and coherence in recruitment. Programs may also need to reassess the number of positions offered, strengthen safety and ergonomics, and modernize interview and selection practices.

### Limitations of the study

This study should be interpreted in the context of a few methodological limitations. The analysis depended on self-reported information that is inherently susceptible to recall errors and subjective interpretation. Although it was an anonymous survey, we believe most applicants relative to the total eligible cohort did not respond to avoid any potential conflict of interest. Moreover, the findings reflect a single match. This study did not collect demographic information such as age, sex, race/ethnicity, or training region, limiting the ability to assess subgroup differences in applicant experiences and perceptions. The survey did not capture exact match cycle participation for all respondents, limiting the ability to provide a detailed cycle-specific breakdown. These limitations underscore the need for longitudinal, multicenter investigations integrating both applicant-reported and program-level data to more comprehensively characterize evolving trends within the IC fellowship recruitment landscape.

## Conclusion

IC remains a demanding but profoundly rewarding career path. Its ability to attract and retain talent will depend on balancing the specialty’s clinical impact and addressing challenges proactively to ensure that IC continues to thrive as both a profession and a vital component of cardiovascular care. Overall, the dominant message from the applicants who participated in the IC NRMP match process was that the match should be consistent, transparent, and fair, with all programs fully committed to the process.

## CRediT authorship contribution statement

**Naseer A. Khan:** Supervision, Software, Resources, Methodology, Data curation, Conceptualization. **Najam U. Saqib:** Software, Methodology, Investigation, Data curation, Conceptualization. **Eva Meunier:** Conceptualization. **Dean J. Kereiakes:** Data curation, Conceptualization. **Luay Alalawi:** Data curation, Conceptualization. **Said Hajouli:** Writing – review & editing, Writing – original draft, Visualization, Validation. **Muhammad Saleem:** Data curation, Conceptualization. **Abubakar Nazir:** Funding acquisition, Formal analysis, Data curation, Conceptualization.

## Declaration of competing interest

The author declared no conflicts of interest.
