# Supplementary material for: Matched and Measured: An In-depth Look at the Interventional Cardiology Fellowship Match—Fellows’ Perspective
Source: J Soc Cardiovasc Angiogr Interv. 2026 Jun 11;5(9):105454. doi: 10.1016/j.jscai.2026.105454 (PMC13591476; doi:10.1016/j.jscai.2026.105454)
Supplement: Supplementary file 1 [file mmc1.docx]

Participants were asked to complete the following questions:

1. What is your current status?
   (e.g., applicant to IC Match, current fellow, structural fellow)
2. How many Interventional Cardiology programs did you apply to?
   (<10, 10–20, 20–30, >30)
3. How many interview invitations did you receive?
   (1–5, 6–10, 11–15, >15)
4. How many interviews did you attend?
   (1–5, 6–10, 11–15, >15)
5. How many programs did you rank on your rank list?
   (1–5, 6–10, 11–15, >15)
6. Did you accept a prematch offer?
   (Yes/No)
7. Did you prefer internal or external programs?
   (Internal/External/Both)
8. What factors influenced your rank list?
   (Select all that apply: procedural volume and complexity, program reputation, mentorship/recommendations, geographic location, coronary/peripheral/structural exposure, program culture/support)
9. What was the biggest challenge during the application cycle?
   (e.g., limited program information, communication issues, interview scheduling, stress/uncertainty)
10. What interview model do you prefer?
    (Virtual/Hybrid/In-person)
11. Do you feel satisfied with the transparency in the IC application process?
    (Yes/No/Prefer not to answer)
12. What changes would you recommend for the IC fellowship Match process?
    (Open-ended)
